# Must We Still Be Worried About Multiple Arteries in Kidney Transplantation?

**DOI:** 10.5812/numonthly.4928

**Published:** 2012-12-15

**Authors:** Cristóbal Moreno-Alarcón, Gerardo Server-Pastor, Pedro Ángel López-González, Pedro López-Cubillana, José Carlos Ruiz-Morcillo, Gloria Doñate-Iñíguez, Edgar Humberto Olarte-Barragán, Guillermo Antonio Gómez-Gómez

**Affiliations:** 1 Department of Urology, University of Murcia, University Hospital Virgen de la Arrixaca, Murcia, Spain

**Keywords:** Arteries, Kidney Transplantation, Outcome Assessments

## Abstract

**Background:**

Multiple renal arteries in kidney grafts have been associated with an increased rate of vascular and urologic complications. Our objective is to compare the outcome of transplanted patients who receive a single pedicle kidney with those who receive a multiple arterial graft.

**Objectives:**

To find our the differences in the outcome and complications in patients undergoing kidney transplantation with one single artery or with multiple arteries.

**Patients and Methods:**

We analyzed 147 kidney transplantations, (all performed in our hospital over a 3 year period). population divided into two groups: group A for those who presented with only one renal artery, or group B for those with more than one renal artery. Homogeneous vascular reconstructions and implantation rules were applied in all patients. We compared the rates of renal failure between the two groups, urinary and vascular complications, patient and graft survivals and the levels of creatinine clearance during the first year of post-transplantation.

**Results:**

No significant differences were found between the two groups regarding to the values analyzed.

**Conclusions:**

As many other authors, we do believe that the presence of multiple renal arteries in kidney grafts should not be considered as a predictive factor of transplantation failure.

## 1. Background

The rate of patients with end-stage renal disease is increasing everyday (1). Kidney transplantation is the best treatment for most of these patients and consequently the number of patients who are waiting for a graft is rising (2). This means that what were contraindications in the past are nowadays just one more challenge to be overcome (3). Studies have shown that anatomical anomalies in donor grafts, extraction injuries, receptor diseases or donor age should not be reasons for rejecting a renal graft. However, the concept of “marginal graft” is growing more flexible every day (4). In Spain, living-donor grafts are not standard, so cadaveric donors are still the main source for transplantation. At the time of extraction, we can expect to find several anomalies that we could have had prior knowledge of, like in living-donor donation, and try to avoid them (for example choosing the opposite kidney), but in deceased donation grafts should be managed to make transplantation possible for the largest number of recipients. But do we really believe those multiple-arteries grafts will have a poorer outcome?

## 2. Objectives

Our goal in this study is to find differences in the outcome and complications of patients with only one artery or with multiple arteries that led us to think in worst, better or equal results in kidney transplantation.

## 3. Patients and Methods

Between January 2006 and December 2008 147 cadaveric-donor kidney transplantations were performed in university hospital Virgen de la Arrixaca by the authors of this paper. We evaluated all of cases, including those grafts that were rejected for some reason at the time of the implantation (for instance, vascular complications). After cadaveric extraction (most of which were multiorganic) bench surgery was carried out and implantation of the graft performed, preferably in the right iliac fossa, with end-to-side anastomosis of the graft main vessels to the external iliac artery and vein avoiding atheromatous plaques. Both anastomoses were performed with two halves of running non-absorbable monofil 6x0 or 5x0 sutures. Extravesical ureteroneocystostomy (Lich-Gregoir technique) with double-J stent was used for urinary reconstruction. All patients received the standard triple drug immunossuppression.

Mean patient age was 50.9 ± 12.24 years (range 18-75 years) and 46.66 ± 16.42 years (range 14-75 years) for the donors; 96 (65.3%) were males and 51 (34.7%) females.

Thirty-three grafts (22.4%) had multiple arteries (group B) and the rest, 114 (77.6%), had one single artery (Group A). Regarding bench reconstructions, when there were polar arteries of less than 2 mm in diameter they were ligated (4 patients). If the distance between the two main arteries (more than 2 mm in diameter) was less than 2 cm, a common aortic patch had been used (in 21 patients). However, if it was greater than 2 cm, we carried out side-to-side (pantaloon) anastomosis (4 patients) when the caliber was of equal size or sequential anastomosis in recipient (5 patients) when the calibers were very different. Only two grafts had three arteries, one of them was ligated because of its small caliber, and bench surgery was performed as in double artery grafts. One graft had 4 arteries, so one polar artery was ligated, two were converted to a single arterial vessel after bench reconstruction and 2 end-to-side anastomosis were performed in the external iliac artery. This patient suffered extensive external iliac vein thrombosis when we removed the vessel clamp and transplantectomy was performed.

To evaluate the outcome of transplantation we recorded the following factors in the first year after surgery: (a) patient and (b) graft survival; (c) the incidence of vascular complications (renal artery and vein thrombosis and renal artery stenosis); (c) urinary complications (ureteral stenosis and leak); (d) delayed graft function (DGF), that was defined as when serum creatinine levels increased or remained unchanged after 5 five days post-transplantation, and the levels of creatinine clearance at one, two, three, six, nine and twelve months.

The differences between the groups of patients (A and B) were analyzed using SPSS 10 software: t-student test for the means of levels of creatinine clearance, Chi-Square test for the complications rates and Kaplan-Meier plots for the graft and patient survival.

## 4. Results

### 4.1. Graft and Patient Survival

The one year graft survival rate was 87.5%; 15 patients lost the graft function (due to surgical causes such as vascular complications with immediate transplantectomy or medical causes such as rejection). Patient survival was 95.5%; six patients died in this period. No statistically significant differences were found between groups A and B (Table 1).

**Table 1:** Complication Rates During the Postoperative Time.

Complication	No. of Patients	Incidence, (%)	Group A/Group B, No.(%)	*P* value
**DGF**	38	25.9	31 (27)/7 (21)	0.495
**Urologic**	22	14.9	18 (16)/4 (12)	0.591
**Stenosis**	8	5.4	7 (6)/1 (3)	-
**Leak**	14	9.5	11 (9.7)/3 (9)	-
**Vascular**	5	3.4	4 (3.5)/2 (6)	0.344
**Arterial thrombosis**	1	0.7	0 (0)/1 (3)	-
**Venous thrombosis**	1	0.7	1 (0.8)/0 (0)	-
**Arterial stenosis**	3	2	2 (1.7)/1 (3)	-

Abbreviation: DGF; delayed graft function

### 4.2. Delayed Graft Function

The incidence of DGF in the 147 patients was 25.9%; 27% in the group A and 21% in the group B and was not significantly different.

### 4.3. Vascular Complications

We only found one case (0.7%) of renal artery thrombosis, one case (0.7%) of renal vein thrombosis and three cases (2%) of renal artery stenosis. The case of artery thrombosis occurred in the patient who was transplanted using the graft with four arteries. Transplantectomy was performed in both cases of vascular thrombosis and the cases of stenosis were successfully treated by balloon angioplasty. No statistically significant differences were found between groups A and B.

### 4.4. Urologic Complications

Eight cases (5.4%) of ureteral obstruction that were treated by balloon ureteroplasty, with only one case of restenosis that required surgery (reimplantation of the ureter into the bladder) had been found. Fourteen patients (9.5%) suffered anastomotic or bladder leakage and urgent reimplantation was performed on all of them. There were no statistically significant differences between both groups.

### 4.5. Creatinine Clearance

No significant differences between groups A and B were found with respect to creatinine clearance at 1, 2, 3, 6, 9 and 12 months. It could be concluded that in the first year renal function was similar in patients with a single-artery graft compared to those with a multiple-artery graft (Table 2).

**Table 2:** Creatinine Clearance Levels

Postoperative Moment, mo	Mean (mL/min)		*P* value
	Group A	Group B	
**1 **	71.07	69.35	0.832
**2 **	70.85	77.52	0.333
**3 **	76.68	81.13	0.538
**6**	82.69	87.03	0.684
**9**	84.92	85.72	0.926
**12**	85.32	92.21	0.428

## 5. Discussion

Throughout the cadaveric extraction the surgeon could find anatomic anomalies that are not uncommon. In fact, Harrison et al. revised arteriographies of future living-donors and concluded that 25% of people had multiples arteries and less than 50% of them had the simplest form of renal vascular pedicle (5). Other authors reported vascular anomalies rates of 28% (6). Prior the nineties, several studies reported a higher rate of complications and acute tubular necrosis when multiple artery grafts were used (7-9). Consequently, it was customary for surgeons to reject grafts found with two or three arteries during extraction because they were problematic to manage in the operating room and after the operation.

Fortunately, after those discouraging studies, due to the long hospital waiting list for deceased donor grafts and the peak in the number of living donors, a larger series of patients were analyzed with very different statistical results. Authors of these studies did not find differences in the outcomes when comparing the patients who received a multiple artery graft with those who received a single pedicle kidney (1, 10-16). Some authors even concluded that every anomaly or injury in the extraction had to be overcome in bench surgery (4). Vascular reconstruction techniques were better defined and they sometimes varied considerably between surgical teams, but it became conceivable to contrast the results of each technique and to draw conclusions about how to improve vascular reconstructions.

As a matter of fact, we cannot deny that the existence of anatomic anomalies in renal grafts is an added difficulty. They need to be dealt with by an experienced team in vascular reconstruction and involve a higher cold and warm ischemic time that can be improved by using the common aortic patch (Carrel patch). Unfortunately, this technique is not possible in living donation. However this increase in ischemic time is not enough to damage the graft if it is adequately perfused and cooled (16).

One limitation of our study could be the monitoring duration (one year). We do believe that the classic complications associated with vascular anomalies in kidney transplantation (acute tubular necrosis, urinary obstructions or leaks, vascular thrombosis) are usually occur early; only arterial stenosis tends to happen later and is usually diagnosed between 6 months and 3 years post-transplantation (11). The management of the urinary and the arterial stenosis should be primarily percutaneous, which leads to good results and low morbidity (12). One important aspect of our study is that all the surgeons who performed the transplantation used the same vascular reconstruction techniques and they all used the external iliac vessels, which means we can consider our methods to be more homogeneous than other prior studies.

Like many other authors we cannot conclude that the use of multiple artery grafts is associated with a poorer outcome. Consequently, it is currently unacceptable to reject organs because of this anomaly and as a result more patients can benefit from the best treatment for the end-stage kidney disease. More studies are necessary in order to shed the light on the subject.
